# Differences in plant- and animal-based protein sources consumed across socioeconomic groups in The Netherlands and their associated environmental impact

**DOI:** 10.1007/s00394-026-03940-w

**Published:** 2026-03-17

**Authors:** Hector A. Lopez Mariaca, Christa Blokhuis, Yinjie Zhu

**Affiliations:** 1 Consumption and Healthy Lifestyles Chair Group, Wageningen University and Research, Hollandseweg 1, 6706 KN Wageningen, The Netherlands; 2 Information Technology group, Wageningen University and Research, Hollandseweg 1, 6706 KN Wageningen, The Netherlands; 3 National Institute for Public Health and the Environment, Antonie van Leeuwenhoeklaan 9, 3721 MA Bilthoven, The Netherlands

**Keywords:** Socioeconomic status, Food consumption, Dietary proteins, Greenhouse effect, Land use, Water use

## Abstract

**Purpose:**

This study explored the differences in sources of protein (SOP) consumed across socioeconomic groups in the Netherlands and their environmental impact.

**Methods:**

Using data from the Dutch National Food Consumption Survey 2019–2021, 1746 participants aged 18–79 years were included. Socioeconomic status (SES) was determined by education level. Protein sources included nuts and seeds, dairy, red meat, poultry, processed meats, fish, legumes, meat substitutes, and eggs. Environmental impact (EI) was assessed using data from life cycle assessment (LCA), considering greenhouse gas (GHG) emissions, land use (LU) and water use (WU). Dietary intake was estimated using two non-consecutive 24-h dietary recalls and reported in g/day; fish consumption was converted to g/w for this study. Linear and logistic regressions were applied to explore the associations between SES (predictor variable) and SOP and EI indicators (response variables).

**Results:**

Compared to the high SES group, individuals with low SES consumed more red meat, processed meat, and dairy (*p* for trend < 0.05 for all), and less nuts and seeds (*p* < 0.001), fish (*p* = 0.004). They were less likely to consume meat substitutes (*p* < 0.001). No significant differences were found for poultry, eggs, and legumes consumption. Individuals with low SES showed greater total GHG emissions and LU but lower WU (*p* < 0.05 for all). Across all SES groups, animal-based protein sources (ABPs) contributed the most to GHG emissions, LU and WU, while plant-based protein sources (PBPs) contributed minimally.

**Conclusions:**

Individuals with low SES tend to consume more ABPs, and were associated with greater GHG emissions and LU, whereas total WU was higher among individuals with high SES, due to a greater consumption of water-intensive PBPs, such as nuts and seeds. These findings highlight socioeconomic disparities in dietary patterns and environmental impact that may be relevant for future strategies toward healthier and more sustainable diets.

**Supplementary Information:**

The online version contains supplementary material available at 10.1007/s00394-026-03940-w.

## Introduction

Socioeconomic status (SES) has been associated with inequalities in dietary patterns in The Netherlands. Dietary practices of low SES groups are often characterized by a low intake of food groups that are associated with beneficial health outcomes (i.e. fruits and vegetables), and a high intake of food groups that are associated with adverse health outcomes (i.e. processed meats and sugar-sweetened beverages) [1–3]. SES inequalities in dietary intake can be attributed to the differences in health and nutrition knowledge, affordability, and accessibility of healthy foods [3–5]. Disparities in food consumption across SES groups may directly impact total dietary protein intake. Dietary protein intake is relevant for human nutrition and health due to their roles in tissue-building, regulating metabolic pathways, and immune system activity [6, 7]. The dietary protein can be derived from both animal- and plant-based sources, with meat, poultry, dairy, eggs, and fish being common animal-based protein sources (ABPs), and legumes, meat replacements (like tofu, tempeh, and meat substitutes), and nuts being common plant-based protein sources (PBPs).

Consumption of different sources of protein (SOP) has implications for human and planetary health [8]. Diets high in ABPs, especially red and processed meat, have been associated with increased risks of mortality, type 2 diabetes mellitus (T2DM), stroke, and different types of cancer (i.e. colorectal cancer) [9–12]. Diets high in ABPs have also been associated with high environmental impact (EI) due to the high demand of natural resources required for their production. The livestock sector accounts for 12–18% of total greenhouse gas (GHG) emissions and utilizes approximately 10% of the estimated annual water flows [8, 13]. Additionally, livestock occupies 26% of ice-free land, while 33% of croplands are destinated to livestock feeding [8, 13]. Conversely, diets predominantly consisting of PBPs have been associated with reduced risks of all-cause mortality, T2DM, cardiovascular disease (CVD), and different types of cancer (including breast, pancreatic, and prostate cancer) [14, 15]. These protective effects are likely due to the high content of fiber, vitamins and antioxidants in PBPs [16]. These plant-based dietary patterns have also shown the potential to reduce diet-related GHG emissions and land use (LU) by around 70–80%, and 50% of water use (WU) compared to western diets characterized by a high content of ABPs. This lower EI has been attributed to fewer resources required to produce PBPs compared to ABPs [8, 17, 18].

In the Netherlands, the government is actively promoting healthier and more sustainable diets by encouraging higher consumption of plant-based options and reducing consumption of animal-based products [19]. To support an equitable protein transition, it is essential to understand how protein consumption patterns, and their associated EI, differ across SES groups. While some studies have explored this association, most of the existing evidence focuses primarily on GHG emissions [3, 20–22].

This study contributes to the current evidence by examining the consumption of different SOP and their associated EI for multiple indicators: GHG emissions, LU and WU across SES groups. This broader approach aims to provide a more comprehensive understanding of potential disparities between SES groups and inform tailored strategies to achieve more inclusive healthy and sustainable diets in The Netherlands.

## Materials and methods

### Study design and study population

A cross-sectional study was conducted using data from the Dutch National Food Consumption Survey (DNFCS) 2019–2021 [23], which included a representative sample of 3570 Dutch children and adults aged 1–79 years. The survey aimed to provide insight into dietary habits in the Dutch population. Data was collected by means of a general questionnaire and two non-consecutive 24-h dietary recalls. Only participants with complete data for both 24-h dietary recalls were included in the original dataset. For representativeness, reported values in the dataset were weighted for demographic factors (such as gender, age, education level, urbanization, etc.), season of data collection, and weekday-weekend combinations of the 24-h dietary recalls. All analyses were conducted on weighted data. A full explanation and description of the DNFCS 2019–2021 is detailed elsewhere [23]. For this study, the target population for analysis comprised 1746 adults aged between 18 and 79 years. Children and adolescents were excluded due to specific dietary requirements and reported parental influence on eating behaviors [24, 25].

### Socioeconomic status and other covariates

Education level, sex, age, and several lifestyle factors such as Body Mass Index (BMI), physical activity, smoking status, and alcohol consumption were self-reported. As information on income or occupation was not available in the DNFCS 2019–2021, educational level was used as a proxy for socioeconomic status (SES). SES was indicated by the highest educational level indicated by participants and classified by the National Institute for Public Health and the Environment (RIVM) into three categories: low education (primary education, lower vocational education, advanced elementary education), middle education (intermediate vocational education, and higher secondary education), and high education (higher vocational education and university). BMI was classified into 4 categories: Underweight (< 18.5 kg/m^2^), Healthy weight (18.5–24.9 kg/m^2^), Overweight (25–29.9 kg/m^2^), and Obesity (≥ 30 kg/m^2^) according to WHO classification [26]. Total physical activity (PA) was classified into 3 categories based on total days per week with at least sufficient moderately intensive activity as follows: Inactive: < 1 day/week, semi-active: 1–5 days/week, and normally active: ≥ 5 days/week, considering 150 min of moderate-intensity activities per week including commuting, leisure time (walking, biking, swimming, fitness), household activities and work. Smoking was defined as current use of tobacco. Alcohol consumption was defined as current consumption of alcohol beverages.

### Dietary assessment

Dietary intake data were collected by trained dietitians using two non-consecutive 24-h dietary recalls, with a period of about four weeks in between. The dietitians used the GloboDiet system to collect the data and standardize the interviews [23]. Food composition data from the Dutch Food Composition Database were linked to the consumption data for the calculation of energy and nutrient composition (NEVO-online 2021) [23]. According to the GloboDiet classification, 18 main food groups were identified [23]. For this study nine SOP were considered and divided into two groups according to their source for further analysis: ABPs (animal-based protein sources) including dairy (milk and milk beverages, yogurt, cheese), red meats (beef, veal, pork, mutton, horse, goat, and rabbit), poultry (chicken, turkey, duck, goose), processed meats, fish (fish and seafood), and eggs; and PBPs (plant-based protein sources) including legumes, nuts and seeds, and meat substitutes. Food consumption was reported in g/day. Fish consumption was converted to g/week for analysis in this study. Given the low consumption reported for legumes, meat substitutes, and eggs, binary variables (yes/no) were created for these SOP to indicate whether any amount of consumption was reported, to explore the number of consumers across SES.

### Environmental impact

To evaluate the EI of SOP consumed, we utilized existing data from the RIVM. This data, detailing the EI of various food groups is reported in the “Database for the Environmental Impact of Food Products” (Database Milieubelasting voedingsmiddelen) [27]. The EI of common foods consumed in The Netherlands was determined through a life cycle assessment (LCA), a technique that assesses environmental loads derived from all stages of a product’s life, including extraction of the raw material, production use, and disposal [28]. Information about the lifecycle of food products was stored in the “Life Cycle Inventory” (LCI). Using the LCA software SimaPro (version 9.0) with the impact assessment model ReCiPe 2016, the LCI was translated into LCA data for six EI indicators. Detailed information about how LCA was developed can be found elsewhere [27].

For this study, GHG emissions, LU, and WU were selected as indicators of EI as they are major contributors to the environmental burden from food production and consumption [8, 29–31], and reflect considerable variability between food groups [32].

For each participant, GHG emissions (kg CO_2_ eq/d), LU (m^2^ a crop eq/d), and WU (L/d) were calculated based on reported consumption of the selected SOP (including legumes, nuts and seeds, meat substitutes, dairy, red meats, poultry, processed meats, fish, and eggs) using data from the 24-h dietary recalls reported by participants in the DNFCS 2019–2021 (Supplementary Table S1). Values were then averaged to estimate EI at the individual level. WU was originally reported in m^3^ by the RIVM and converted to liters (L) for data analysis.

### Statistical analysis

Statistical analyses were performed using RStudio, version 4.3.2. Descriptive statistics of the study population were presented across SES groups. The normality of variables was assessed according to the Shapiro-Wilk test and histograms. Normally distributed data were presented as means (standard deviation (SD)), categorical variables as numbers (percentage (%)), and non-normally distributed data as medians (interquartile range (IQR). The differences in SOP consumed across SES groups were analyzed using a one-way ANOVA test for normally distributed data, Kruskal-Wallis ANOVA test for non-normally distributed data and the Chi-Square test for categorical variables.

To analyze differences in EI associated with SOP consumed across SES groups, GHG emissions, LU, and WU were calculated per participant based on their reported consumption in the DNFCS 2019–2021. Total GHG emissions, LU, and WU were then calculated and stratified by ABPs and PBPs; ratios (PBPs EI/Total EI) were also calculated. A Kruskall-Wallis ANOVA test was used to analyze differences in the medians of total GHG emissions, LU, and WU, EI derived from ABPs and PBPs, and PBPs EI/Total EI ratios across SES groups.

Multivariate linear regression analyses were applied to investigate the association between SES groups, SOP consumed, and EI indicators (GHG emissions, LU and WU) using weighted data to ensure population representativeness. SES was entered as a categorical variable, using the high SES as the reference category, whereas SOP consumed, and EI indicators were managed as continuous variables. A multivariate logistic regression analysis was used to explore the association between SES and consumers of legumes, meat substitutes and eggs, managed as binary variables (yes/no). For all regression analyses, three different models were used: Model 1 adjusted for age and sex; Model 2 adjusted for age, sex, caloric intake and BMI; and Model 3 adjusted for age, sex, calorie intake, BMI, smoking, alcohol consumption, and physical activity. Model 3 was considered the primary model for interpretation. The effect modification of sex on the association between SES and SOP consumed and EI indicators was also investigated. Statistically significant values were considered as *p*-value ≤ 0.05. Finally *p*-values for trend were calculated by fitting SES as an ordinal variable in the models.

## Results

### Study population characteristics and dietary intake

The sociodemographic characteristics, lifestyle factors, and dietary intake are denoted in Table 1. The average age of the subjects in the study population was 55 ± 16 years. The low SES group had a lower proportion of men, compared to middle and high SES groups. The prevalence of overweight and obesity was higher in individuals with lower SES. In the low SES group, more participants were current smokers, consumed alcohol, and were physically inactive.

The average total calorie and total protein intake were 2059.3 ± 607.3 kcal/d and 80.6 ± 24.9 g/d respectively. No difference was found between total protein intake across SES groups; however, with the increase of SES, total calorie and plant-based protein intake increased (*p* ≤ 0.001) while animal-based decreased (*p* = 0.002). On average, plant-based protein intake was 5.2 g/d higher in the high SES group compared to the low SES group, whereas animal-based protein intake was 4.3 g/d lower.

The median intake of nuts and seeds was 7.4 g/d higher in the high SES group (*p* ≤ 0.001), while red and processed meat was 16 g/d higher in the low SES group (*p* ≤ 0.001 for both). Median fish intake was 0 g/w across SES groups, but the distribution differed significantly (*p* = 0.015), indicating higher consumption among individuals among the high SES group. Overall, one out of 10 participants consumed meat substitutes, and the proportion of meat substitutes consumers was double (14.5%) among the high SES group compared to the low and middle SES groups (6.1 and 7.6% respectively). No significant differences across SES groups were found for legumes, dairy, poultry, and eggs.

**Table 1:** Characteristics of the total study population and SOP consumed

	Total (*n* = 1746)	Low SES (*n* = 445)	Middle SES (*n* = 632)	High SES (*n* = 669)	*p*
*Demographics*					
Age (years)	55 ± 16	61 ± 15	53 ± 16	53 ± 15	0.001
Male, n (%)	879 (50.3%)	195 (43.8%)	315 (49.8%)	369 (55.2%)	< 0.001
*Lifestyle*					
BMI (kg/m^2^*)*	26.6 ± 5.1	27.7 ± 5	27.1 ± 5.6	25.5 ± 4.3	< 0.001
Underweight, n (%)	24 (1.4%)	4 (0.9%)	10 (1.6%)	10 (1.5%)	< 0.001
Healthy weight, n (%)	716 (41.0%)	132 (29.7%)	241 (38.1%)	343 (51.3%)	
Overweight, n (%)	631 (36.1%)	191 (42.9%)	228 (36.1%)	212 (31.2%)	
Obesity, n (%)	375 (21.5%)	118 (26.5%)	153 (24.2%)	104 (15.5%)	
*Current smoking, n (%)*				< 0.001	
Yes	243 (13.9%)	82 (18.4%)	101 (16.0%)	60 (9.0%)	
*Current alcohol consumption,* *n (%)*					< 0.001
Yes	1265 (72.5%)	288 (64.7%)	452 (71.5%)	525 (78.5%)	
Physical activity, *n (%)*					0.04
Inactive	55 (3.2%)	21 (4.7%)	21 (3.3%)	13 (1.9%)	
Semi-active	373 (21.4%)	101 (22.7%)	141 (22.3%)	131 (19.6%)	
Normally-active	1318 (75.5%)	323 (72.6%)	470 (74.4%)	525 (78.5%)	
*Dietary intake*					
Calorie intake (kcal/d)	2059.3 ± 607.3	1976.8 ± 565.5	2062.4 ± 617.9	2111.2 ± 618.9	0.001
Total protein (g/d)	80.6 ± 24.9	79.8 ± 23.6	81.2 ± 26.6	80.7 ± 24.2	0.653
Plant-based protein (g/d)	31.6 ± 11.6	28.5 ± 10.4	31.4 ± 11.3	33.7 ± 12.2	< 0.001
Animal-based protein (g/d)	49 ± 21	51.2 ± 19.6	49.8 ± 22.1	46.9 ± 20.7	0.002
Nuts and seeds (g/d)	1.2 (0–21.4)	0 (0–15)	0 (0–21.4)	7.4 (0–25)	< 0.001
Dairy (g/d)	265.4 (123.4–441.1)	281.9 (138.1–464)	268.7 (115–444)	256.7 (120.1–421.7)	0.251
Red meat (g/d)	0 (0–46.4)	16.0 (0–56.5)	6.26 (0–49.1)	0 (0–37.5)	< 0.001
Poultry (g/d)	0 (0–23.5)	0 (0–21)	0 (0–28.1)	0 (0–21)	0.13
Processed meat (g/d)	33.4 (8–67.5)	41 (13.5–78.5)	37 (9.7–67.3)	25 (0–55.5)	< 0.001
Fish (g/w)	0 (0–106.7)	0 (0–0)	0 (0–84.6)	0 (0–197)	0.015
Legumes n (%) Yes	205 (11.7%)	53 (11.9%)	66 (10.4%)	86 (12.9%)	0.398
Meat substitutes n (%) Yes	172 (9.8%)	27 (6.1%)	48 (7.6%)	97 (14.5%)	< 0.001
Eggs n (%) Yes	778 (44.6%)	192 (43.2%)	284 (44.9%)	302 (45.1%)	0.783

Values are mean + standard deviation for normally distributed data, median (interquartile range) for non-normally or skewed continuous variables, and numbers (percentages) for categorical data. Data for legumes, meat substitutes, and eggs, is shown in proportion of consumers due to low reported consumption

### Differences in EI across SES groups

**Fig. 1:**
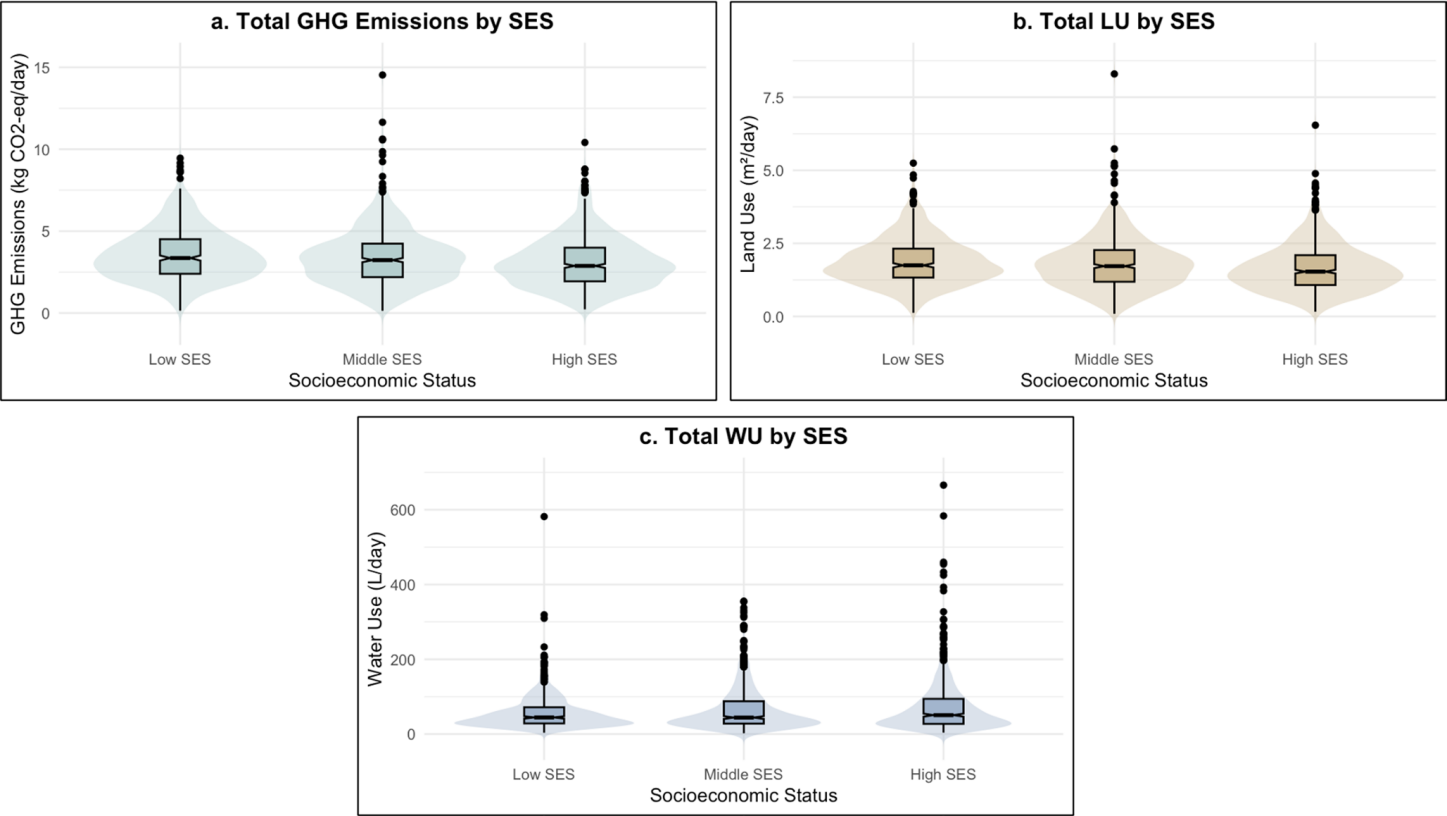
Environmental impact from SOP across SES groups. **a** Shows the Total GHG emissions derived from SOP consumed across SES. **b** Shows the Total LU derived from SOP consumed across SES. **c** Shows total WU derived from SOP consumed across SES. Violin plots show data distribution density. Whiskers show minimum and maximum range, excluding outliers which are shown as dots, and represent values more than 1.5 times 1st and 3rd quartiles

The total EI and SOP-specific EI for total population and across SES groups are presented in Supplementary Table S2. In the study population, the median daily GHG emissions, LU, and WU were 3.13 (2.16–4.20) kg CO_2_ eq/d, 1.66 (1.18–2.21) m^2^ crop eq, and 46.94 (27.72–86.76) L per person, respectively. Significant differences in total GHG emissions and LU were observed across SES groups (*p* < 0.001 for both). Median values of total EI across SES groups is shown in Fig. 1. The low SES group showed the highest median total GHG emissions (3.36 kg CO_2_ eq/d) (Fig. 1a) and LU (1.75 m²/d) (Fig. 1b), followed by the middle and high SES groups. In contrast, total WU was higher among the high SES (50.69 L/d), while the low SES group had the lowest (44.45 L/d) (Fig. 1c). Differences in total WU across groups were not statistically significant (*p* = 0.126).

Regarding SOP-specific EI, GHG emissions, LU and WU derived from ABPs were higher among low SES groups, whereas those derived from PBPs were higher among high SES groups (*p* < 0.001 for all) (Supplementary Table S2). For instance, GHG emissions from ABPs were 0.51 kg CO_2_ eq/d higher in the low SES group compared to the high SES group (3.32 vs. 2.81 kg CO_2_ eq/d), while GHG emissions from PBPs were 0.05 kg CO_2_ eq/d higher in the high SES group (0.06 vs. 0.01 kg CO_2_ eq/d). Similarly, LU and WU from PBPs were 0.11 m²/d and 18.73 L/d higher, respectively, in the high SES group. Furthermore, the ratios of GHG emissions, LU and WU derived from PBPs to their respective totals, were higher in high SES groups (*p* < 0.001 for all), suggesting a greater relative contribution in this group, while ABPs had a relatively larger contribution in the low SES group (Supplementary Table S2).

### Association between SES groups with SOP consumed

The association between SES and different SOP consumed is presented in Table 2. Compared to individuals with high SES, those with low SES showed a lower intake of total plant-based protein, nuts and seeds (both *p* for trend < 0.001) and fish (*p* for trend = 0.004), but showed a higher intake of total protein intake β (ΣΕ) 2.41 (1.05), *p* = 0.02), dairy (33.37 (14.81), *p* = 0.024), red meat (9.84 (2.62), *p* < 0.001) and processed meat) 18.10 (2.91), *p* < 0.001) after adjusting for age, sex, calorie intake, BMI, smoking, alcohol consumption and PA. Sex modified the association of SES with total protein intake and total animal-based protein (*p*_interaction_ = 0.018 and 0.027 respectively) with a higher association magnitude found among females (Supplementary Table S3). No significant association was found between SES with total calorie intake (*p* > 0.05).

The association between SES and the consumption of legumes, meat substitutes and eggs was tested through logistic regression analysis (Table 3). Compared to individuals with high SES, those with low SES were significantly less likely to consume meat substitutes (OR: 0.55; 95% CI: 0.42–0.72; *p* < 0.001), whereas no significant association was observed between SES with legumes and eggs consumption (*p* > 0.05). Sex modified the association of SES with consumption of legumes (*p*_interaction_ = 0.004), where a higher negative association magnitude was also found among females (Supplementary Table S4).

**Table 2:** Association of socioeconomic groups with the consumption of different SOP

	High SES Reference	Middle SES		Low SES		*P* for trend
		β (ΣΕ)	*p*	β (ΣΕ)	*p*	
*Calorie intake (kcal/d)*						
Model 1	1	− 24.16 (30.29)	0.425	− 48.74 (34.13)	0.154	0.149
Model 2	1	− 5.71 (30.43)	0.851	− 26.19 (34.34)	0.445	0.463
Model 3	1	− 3.48 (30.47)	0.909	− 20.81 (34.68)	0.548	0.567
*Total protein intake (g/d)*						
Model 1	1	1.52 (1.28)	0.234	1.47 (1.44)	0.309	0.025
Model 2	1	1.63 (0.91)	0.075	2.17 (1.03)	0.035	0.027
Model 3	1	1.78 (0.92)	0.053	2.41 (1.05)	0.021	0.015
*Total plant-based protein (g/d)*						
Model 1	1	− 1.99 (0.59)	< 0.001	− 3.64 (0.67)	< 0.001	< 0.001
Model 2	1	− 1.4 (0.46)	0.002	− 2.67 (0.52)	< 0.001	< 0.001
Model 3	1	− 1.28 (0.46)	0.005	− 2.54 (0.52)	< 0.001	< 0.001
*Total animal-based protein (g/d)*						
Model 1	1	3.54 (1.11)	0.001	5.13 (1.26)	< 0.001	< 0.001
Model 2	1	3.06 (0.99)	0.002	4.87 (1.12)	< 0.001	< 0.001
Model 3	1	3.09 (0.10)	0.001	4.99 (1.14)	< 0.001	< 0.001
*Nuts and seeds (g/d)*						
Model 1	1	− 3.37 (1.29)	0.008	− 6.61 (1.45)	< 0.001	< 0.001
Model 2	1	− 2.77 (1.23)	0.024	− 5.61 (1.39)	< 0.001	< 0.001
Model 3	1	− 2.50 (1.23)	0.042	− 5.25 (1.41)	< 0.001	< 0.001
*Dairy (g/d)*						
Model 1	1	9.53 (13.27)	0.472	20.20 (14.95)	0.177	0.174
Model 2	1	12.79 (12.97)	0.324	26.30 (14.64)	0.072	0.071
Model 3	1	17.19 (13.01)	0.187	33.37 (14.81)	0.024	0.023
*Red meat (g/d)*						
Model 1	1	7.29 (2.30)	0.001	11.34 (2.60)	< 0.001	< 0.001
Model 2	1	6.34 (2.29)	0.005	10.43 (2.60)	< 0.001	< 0.001
Model 3	1	5.96 (2.30)	0.009	9.84 (2.62)	< 0.001	< 0.001
*Poultry (g/d)*						
Model 1	1	1.72 (1.78)	0.334	1.66 (2.00)	0.41	0.360
Model 2	1	1.36 (1.79)	0.447	1.30 (2.02)	0.52	0.478
Model 3	1	1.36 (1.81)	0.451	1.28 (2.06)	0.53	0.491
*Processed meats (g/d)*						
Model 1	1	10.73 (2.63)	< 0.001	19.74 (2.96)	< 0.001	< 0.001
Model 2	1	9.10 (2.55)	< 0.001	18.18 (2.88)	< 0.001	< 0.001
Model 3	1	8.91 (2.56)	< 0.001	18.10 (2.91)	< 0.001	< 0.001
*Fish (g/w)*						
Model 1	1	− 21.67 (14.55)	0.136	− 51.83 (16.39)	0.001	0.001
Model 2	1	− 22.45 (14.64)	0.125	− 51.97 (16.53)	0.001	0.001
Model 3	1	− 20.31 (14.73)	0.168	− 48.30 (16.77)	0.004	0.004

Multivariate linear regression analysis. Model 1: Adjusted for age and sex. Model 2: Adjusted for age, sex, calorie intake and BMI. Model 3: Adjusted for age, sex, calorie intake, BMI, smoking, alcohol consumption and PA. SOP (Sources of protein), ΣΕ (Standard error), SES (Socioeconomic status)

**Table 3:** Association of socioeconomic groups with the consumption of legumes, meat substitutes and eggs

		Middle SES		Low SES		*P* for trend
		OR (95% CI)	*p*	OR (95% CI)	*p*	
*Legumes (yes) n (%)*						
Model 1	1	0.80 (0.56–1.12)	0.191	0.98 (0.67–1.42)	0.905	0.732
Model 2	1	0.78 (0.55–1.10)	0.164	0.96 (0.65–1.39)	0.819	0.660
Model 3	1	0.79 (0.56–1.12)	0.193	0.98 (0.67–1.44)	0.926	0.765
*Meat substitutes (yes) n (%)*						
Model 1	1	0.46 (0.31–0.66)	< 0.001	0.39 (0.25–0.29)	< 0.001	< 0.001
Model 2	1	0.50 (0.34–0.72)	< 0.001	0.45 (0.28–0.70)	< 0.001	< 0.001
Model 3	1	0.51 (0.35–0.74)	< 0.001	0.46 (0.28–0.72)	0.001	< 0.001
*Eggs (yes) n (%)*						
Model 1	1	0.99 (0.79–1.23)	0.899	0.87 (0.68–1.11)	0.253	0.284
Model 2	1	0.98 (0.67–1.11)	0.887	0.87 (0.79–1.23)	0.261	0.289
Model 3	1	1.00 (0.80–1.25)	0.992	0.89 (0.69–1.15)	0.361	0.400

Multivariate linear regression analysis. Model 1: Adjusted for age and sex. Model 2: Adjusted for age, sex, calorie intake and BMI. Model 3: Adjusted for age, sex, calorie intake, BMI, smoking, alcohol consumption and PA. OR (Odds Ratios), CI (Confidence intervals), SES (Socioeconomic status)

### Association between SES groups and EI

The association between SES and GHG emissions derived from SOP is shown in Table 4. Compared to the high SES group, the middle and low SES groups were significantly associated with higher total GHG emissions (β (ΣΕ): 0.31 (0.08) and 0.55 (0.09), respectively) after adjusting for age, sex, calorie intake, BMI, smoking, alcohol consumption and PA. This pattern was also similar for GHG emissions derived from ABPs (*p* for trend < 0.001).

On the other hand, the middle and low SES groups showed lower GHG emissions from PBPs compared to the high SES group, with (β (ΣΕ): − 0.03 (0.01) and − 0.04 (0.01) respectively). Similarly, the GHG PBPs/Total GHG ratio was lower in the middle and low SES groups compared to the high SES group. Sex modified the associations of SES with total GHG emissions, ABPs GHG emissions, and GHG PBPs/Total GHG ratio (*p*_interaction_ = 0.031, 0.024 and 0.004 respectively) with a higher association magnitude found among females (Supplementary Table S5).

**Table 4:** Association between SES and GHG emissions derived from SOP across SES groups

	High SES Reference	Middle SES		Low SES		*P* for trend
		β (ΣΕ)	*p*	β (ΣΕ)	*p*	
*Total GHG*						
Model 1	1	0.32 (0.08)	< 0.001	0.53 (0.09)	< 0.001	< 0.001
Model 2	1	0.30 (0.08)	< 0.001	0.52 (0.09)	< 0.001	< 0.001
Model 3	1	0.31 (0.08)	< 0.001	0.55 (0.09)	< 0.001	< 0.001
*GHG ABPs*						
Model 1	1	0.36 (0.09)	< 0.001	0.58 (0.09)	< 0.001	< 0.001
Model 2	1	0.32 (0.08)	< 0.001	0.56 (0.09)	< 0.001	< 0.001
Model 3	1	0.34 (0.08)	< 0.001	0.59 (0.09)	< 0.001	< 0.001
*GHG PBPs*						
Model 1	1	− 0.03 (0.01)	< 0.001	− 0.04 (0.01)	< 0.001	< 0.001
Model 2	1	− 0.03 (0.01)	< 0.001	− 0.04 (0.01)	< 0.001	< 0.001
Model 3	1	− 0.03 (0.01)	< 0.001	− 0.04 (0.01)	< 0.001	< 0.001
*GHG PBPs Ratio*						
Model 1	1	− 0.02 (0.01)	< 0.001	− 0.03 (0.01)	< 0.001	< 0.001
Model 2	1	− 0.02 (0.01)	< 0.001	− 0.02 (0.01)	< 0.001	< 0.001
Model 3	1	− 0.02 (0.01)	< 0.001	− 0.02 (0.01)	< 0.001	< 0.001

Multivariate linear regression analysis. Model 1: Adjusted for age and sex. Model 2: Adjusted for age, sex, calorie intake and BMI. Model 3: Adjusted for age, sex, calorie intake, BMI, smoking, alcohol consumption and PA. GHG (Greenhouse gas) kg CO_2_ eq/d, GHG ABPs (Greenhouse gas emissions derived from Animal-based protein sources), GHG PBPs (Greenhouse gas emissions derived from Plant-based protein sources), GHG PBPs Ratio (GHG PBPs/Total GHG), ΣΕ (Standard error), SES (Socioeconomic status)

The association between SES and LU derived from the consumption of SOP is shown in Table 5. Compared to the high SES group, the middle and low SES groups showed higher total LU(β (ΣΕ): 0.16 (0.04) and 0.28 (0.05), respectively), after adjusting for age, sex, calorie intake, BMI, smoking, alcohol consumption and PA. A similar trend was found for LU derived from ABPs (*p* for trend < 0.001).

In contrast, the middle and low SES groups showed lower LU derived from PBPs compared to the high SES group, (− 0.03 (0.01) and − 0.05 (0.01), respectively). Similarly, the LU PBPs/Total LU ratio was lower in the middle and low SES groups compared to the high SES group. Sex also modified the associations between SES with total LU, ABPs LU, and LU PBPs/Total LU ratio (*p*_interaction_ = 0.031, 0.024 and 0.004 respectively) with a higher association magnitude found among females (Supplementary Table S6).

**Table 5:** Association between SES and LU derived from SOP across SES groups

	High SES Reference	Middle SES		Low SES		*P* for trend
		β (ΣΕ)	*p*	β (ΣΕ)	*p*	
*Total LU*						
Model 1	1	0.17 (0.04)	< 0.001	0.28 (0.05)	< 0.001	< 0.001
Model 2	1	0.16 (0.04)	< 0.001	0.27 (0.04)	< 0.001	< 0.001
Model 3	1	0.16 (0.04)	< 0.001	0.28 (0.05)	< 0.001	< 0.001
*LU ABPs*						
Model 1	1	0.21 (0.04)	< 0.001	0.35 (0.05)	< 0.001	< 0.001
Model 2	1	0.19 (0.04)	< 0.001	0.33 (0.05)	< 0.001	< 0.001
Model 3	1	0.19 (0.04)	< 0.001	0.34 (0.05)	< 0.001	< 0.001
*LU PBPs*						
Model 1	1	− 0.04 (0.01)	< 0.001	− 0.06 (0.01)	< 0.001	< 0.001
Model 2	1	− 0.04 (0.01)	< 0.001	− 0.05 (0.01)	< 0.001	< 0.001
Model 3	1	− 0.03 (0.01)	< 0.001	− 0.05 (0.01)	< 0.001	< 0.001
*LU PBPs Ratio*						
Model 1	1	− 0.04 (0.01)	< 0.001	− 0.06 (0.01)	< 0.001	< 0.001
Model 2	1	− 0.04 (0.01)	< 0.001	− 0.05 (0.01)	< 0.001	< 0.001
Model 3	1	− 0.04 (0.01)	< 0.001	− 0.05 (0.01)	< 0.001	< 0.001

Multivariate linear regression analysis. Model 1: Adjusted for age and sex. Model 2: Adjusted for age, sex, calorie intake and BMI. Model 3: Adjusted for age, sex, calorie intake, BMI, smoking, alcohol consumption and PA. LU (Land Use) m² crop eq/d, LU ABPs (Land Use derived from Animal-based protein sources), LU PBPs (Land Use derived from Plant-based protein sources) LU PBPs Ratio (LU PBPs/Total LU), ΣΕ (Standard error), SES (Socioeconomic status)

The association between SES and WU derived from the consumption of SOP is shown in Table 6. Lower total WU was observed in the middle and low SES groups compared to the high SES group (β (ΣΕ): − 3.84 (3.24) and − 9.04 (3.69) respectively) after adjusting for age, sex, calorie intake, BMI, smoking, alcohol consumption, and PA. Conversely, higher WU derived from ABPs was observed in the middle and low SES groups compared to the high SES group (3.10 (0.70) and 5.19 (0.80) respectively).

The middle and low SES groups demonstrated lower WU derived from PBPs compared to the high SES group, (β (ΣΕ): − 6.94 (3.29) and − 14.23 (3.74), respectively). Similarly, the WU PBPs/Total WU ratio was lower in the middle and low SES groups compared to the high SES group. Sex also modified the association between SES with ABPs WU (*p*_interaction_ = 0.015) showing a higher association magnitude found among females (Supplementary Table S7).

**Table 6:** Association between SES and WU derived from SOP across SES groups

	High SES Reference	Middle SES		Low SES		*P* for trend
		β (ΣΕ)	*p*	β (ΣΕ)	*p*	
*Total WU*						
Model 1	1	− 5.91 (3.50)	0.091	− 12.64 (3.94)	0.001	0.001
Model 2	1	− 4.61 (3.23)	0.154	− 10.16 (3.65)	0.005	0.005
Model 3	1	− 3.84 (3.24)	0.236	− 9.04 (3.69)	0.015	0.014
*WU ABPs*						
Model 1	1	3.36 (0.76)	< 0.001	5.24 (0.86)	< 0.001	< 0.001
Model 2	1	3.02 (0.70)	< 0.001	5.03 (0.79)	< 0.001	< 0.001
Model 3	1	3.10 (0.70)	< 0.001	5.19 (0.80)	< 0.001	< 0.001
*WU PBPs*						
Model 1	1	− 9.26 (3.43)	0.007	− 17.88 (3.90)	< 0.001	< 0.001
Model 2	1	− 7.63 (3.28)	0.020	− 15.19 (3.70)	< 0.001	< 0.001
Model 3	1	− 6.94 (3.29)	0.035	− 14.23 (3.74)	< 0.001	< 0.001
*WU PBPs Ratio*						
Model 1	1	− 0.08 (0.02)	< 0.001	− 0.13 (0.02)	< 0.001	< 0.001
Model 2	1	− 0.06 (0.02)	< 0.001	− 0.11 (0.02)	< 0.001	< 0.001
Model 3	1	− 0.06 (0.02)	0.002	− 0.10 (0.02)	< 0.001	< 0.001

Model 1: Adjusted for age and sex. Model 2: Adjusted for age, sex, calorie intake and BMI. Model 3: Adjusted for age, sex, calorie intake, BMI, smoking, alcohol consumption and PA. WU (Water Use) L/d, WU ABPs (Water Use derived from Animal-based protein sources), WU PBPs (Water Use derived from Plant-based protein sources) WU PBPs Ratio (WU PBPs/Total WU), ΣΕ (Standard error), SES (Socioeconomic status)

## Discussion

This cross-sectional study aimed to investigate the differences in SOP consumption across SES groups and their associated EI by integrating a more comprehensive approach including GHG emissions, LU, and WU, complementing existing research in the Dutch population that mainly focuses on GHG emissions as an EI indicator. Our findings showed that low SES, as determined by education level, was significantly associated with higher intake of total protein, animal-based protein, dairy, red meat, and processed meat, alongside a lower consumption of plant-based protein, nuts and seeds, and fish. Individuals with low SES were also less likely to consume meat substitutes. No significant differences were observed for calorie intake, poultry, eggs, or legumes consumption across SES groups. Regarding EI, the low SES group was associated with higher total GHG emissions and LU but less total WU. Across all groups, ABPs were the main contributors to GHG emissions, LU and WU, whereas the PBPs contributed minimally.

### SOP consumed across SES

Our results indicate that individuals with low SES consume more red and processed meat, and less nuts, seeds, and fish, which is consistent with existing literature [3, 33, 34]. Disparities in food consumption across SES groups have commonly been explained by differences in neighborhood food accesibility, economic constraints, as well as the poor attitude towards healthy eating in low SES and the greater knowledge on healthy food items among high SES groups [35–37]. The likelihood of practicing “healthy and sustainable diets” characterized by higher consumption of PBPs and fish has also been positively associated with higher SES in similar countries. A study conducted in Belgium among Flemish adults, showed that a more plant-based diet was associated with high SES and living in urban areas [38], whereas in Germany, high-income individuals also engaged with more healthy and sustainable diets, eating more plant-based and less animal-based protein sources [39].

Interestingly, we observed a stronger positive association in the intake of animal-based protein among women, which is not consistent with current evidence. Several studies show, in contrast, that men tend to consume higher amounts of animal-based products (such as meat) attributed higher energy intake, masculine identity and conformity with traditional gender roles [40, 41]. A plausible explanation to our findings would be the changes in dietary patterns during COVID lockdowns, where women reported to eat more and found it harder to make healthy food choices compared to men [42]. Another explanation relates to the adjustment for total energy intake. Since women generally have lower total energy intake and lower basal metabolic rate than men, energy adjusted models may influence the direction of the association. Additionally within the lower total energy intake, women may obtain a greater proportion of their calories from animal-based foods, whereas men may consume relatively more high-volume and lower calorie-dense foods such as grains and tubers [23, 43].

Higher consumption of dairy among lower SES groups also aligns with previous findings [44]. Dairy consumption between 2007 and 2010 was higher among high SES groups, however, an overall decreasing trend was observed since 2012, where low SES groups consume higher amount of dairy [44].

Furthermore, we found that individuals with higher SES groups were more likely to consume meat substitutes. This aligns with findings from a cross-national survey conducted in the United Kingdom and The Netherlands that reported a higher proportion of “non-users” in lower SES groups [45]. Different barriers have been identified towards consumption of these products across SES groups. For example, low SES neighborhoods often face fewer options in restaurants, canteens at work/school, plus a decrease in local food shops between 2004 and 2018, limiting access to these products [46, 47]. Additionally, “liking meat” has also been identified as a barrier. The phenomenon called “mere exposure” explains that subjects may require time to develop the acceptance of unfamiliar products [45], where people with higher SES have reported more interest in consuming these substitutes [48]. The theory of planned behavior also supports this, as higher SES groups and neighborhoods tend to exhibit stronger intentions and attitudes toward “healthier behaviors,” such as adopting healthier diets [49]. Pricing has also been considered an important barrier. Meat substitutes tend to be more expensive than their animal-based benchmarks, although actual prices vary depending on the type of product, brand, store and temporary discounts [50]. A study conducted in The Netherlands found that although consumers perceive meat substitutes as healthier than meat, they are still not willing to buy them, likely due to factors such as higher prices and lower palatability [51]. Aligned with this, another study showed that consumers perceived lowering prices of meat substitutes as an effective strategy to reduce meat consumption [46].

The null association between SES and the consumption of legumes, eggs and poultry found in our study does not necessarily align with existing evidence. A study conducted among another Dutch cohort (Lifelines Cohort), reported higher eggs consumption among men in low SES groups, but found no significant differences across SES groups in women. Similarly, legumes consumption showed no significant differences among SES groups but indicated a higher consumption among men compared to women [52]. Although evidence on poultry consumption across SES groups is limited, overall its consumption has remained low since 2016 [3, 23]. Disparities between our study and the Lifelines Cohort findings may be explained by the use of different dietary assessment tools. The DNFCS 2019–2021 used 24-h dietary recall for dietary intake assessment, while the Lifelines Cohort used a Food Frequency Questionnaire (FFQ). It is known that 24-h dietary recall may not fully capture habitual diets, whereas FFQs provides a broader perspective on average food group consumption [53].

### EI across SES

Regarding EI, our study found that low SES groups had higher total GHG emissions and LU but lower WU. These findings are partially in line with limited existing evidence exploring the association of SES with EI derived from SOP. For GHG emissions, previous evidence in The Netherlands shows that high SES groups have healthier diets (including more PBPs), which have been associated with lower GHG emissions [21, 54]. However, a study conducted in four European countries showed contrasting patterns across SES groups, for instance: in the Czech Republic GHG emissions were higher among high SES groups, whereas in France an opposite association was found [55]. In Ireland, a positive association was found between middle SES groups and GHG emissions [56]. Higher GHG emissions have consistently been linked to higher consumption of ABPs compared to PBPs [8, 20, 22], mainly due to the significant contribution of feed for livestock and ruminant enteric fermentation to GHG emissions [57, 58]. In our study this pattern was evident in the low SES group, which showed higher GHG emissions derived from ABPs compared to PBPs. This group also consumed more red meat, processed meat, and dairy; food items known by generating higher amounts of GHG emissions.

Compared to GHG emissions, evidence on the association between SES with WU or LU derived derived from SOP remains limited. Our study showed a higher consumption of ABPs among low SES groups, resulting in more LU derived from ABPs compared to PBPs, which is consistent with existing evidence. A study conducted in 16 European countries found that diets from individuals with low SES required 20% more land compared to diets from people with higher SES [59]. Variation between countries was attributed to agricultural productivity, whereas variation within countries was driven by caloric availability. This study also showed that animal-based products contributed the largest proportion of LU [59]. Red and processed meats have been shown to require significantly more LU than other SOP due to the extensive land needed to grow feed for beef, cattle and pigs [60].

Regarding WU, our study showed that the high SES group consumed more PBPs, contributing to higher WU compared to the low SES group. This aligns with previous research linking healthier diets, categorized as higher in fruits, vegetables, and plant-based proteins, with greater WU in The Netherlands [20]. A scenario analysis conducted in Germany found similar results: vegan and vegetarian diets, required more WU compared to healthy diets that include meat, primarily due to higher consumption of nuts which are PBPs with particularly high water footprint [61]. While these studies did not explore differences in WU across SES groups, higher SES has been consistently associated with healthier dietary patterns [62, 63]. Thus, the increased WU among individuals with high SES in our study is driven not by greater consumption of ABPs, but by a higher intake of high-water-use PBPs such as nuts [20, 64].

### Implications for public health and nutritional advice

Diets of individuals with low SES imposed greater GHG emissions and LU due to their higher consumption of ABPs, particularly red, processed meats, compared to higher SES groups. Given the evidence on the health and environmental implications associated with high consumption of these SOP (especially red and processed meat) [8–12, 20], it remains important to develop and reinforce nutrition policies in The Netherlands that reduce the intake of these ABPs, and promote the consumption of PBPs.

Reducing the consumption of some ABPs may raise nutritional concerns, as red meat is considered a key source of iron, zinc, vitamin B12 [65, 66]. Food policies should encourage the use of plant-based substitutes that support adequate nutrient intake, including nuts, seeds, and legumes due to their high content of protein, fiber, vitamins, iron, calcium and zinc [16, 19, 67]. Soy and pea protein-based substitutes are especially recommended due to their high protein quality [68] as well as formulations combining pea, wheat, and soy protein [69]. Despite concerns about protein adequacy, most substitutes match the average of protein content of their benchmarks [50]. However, special attention should be given to substitutes with high content of salt and sugar [50, 70, 71]. Regarding dairy consumption, although the low SES group reported a relatively higher intake than the high SES group, overall consumption was still below the Dutch dietary recommendation of 2–3 servings per day. Therefore, dairy intake should remain encouraged given its contribution to calcium, vitamin B2, and vitamin B12 intake [72, 73], as well as reported benefits for cardiovascular health and reduced risk of colorectal cancer [74].

Regarding the higher WU observed among the high SES group due to the intake of water-intensive PBPs such as nuts, evidence indicates that more plant-based dietary patterns can still result in overall less water footprint when they replace water intensive ABPs [17, 18]. To support this transition and considering the variability in WU across PBPs, strategies could prioritise plant-based alternatives that require less water, such as legumes [8]. When promoting nuts, attention should be given to those with lower water footprints, including groundnuts and chestnuts [64].

While SES influences dietary choices, individual’s food consumption is shaped by other factors at multiple socio-ecological levels [75]. Structural barriers such as affordability, accessibility and acceptability should be addressed in food policy-making. Strategies such as subsidies for PBPs, (including meat substitutes), food-media campaigns and worksite wellness programs have shown to be effective in the increase in consumption of healthy foods [76, 77]. Further research should also explore how these interventions can be tailored for different SES groups to maximize their impact towards healthier and more sustainable diets.

### Strengths and limitations

This study has several strengths. Firstly, it complements existing evidence on sustainable and healthy diets in the Netherlands [20, 22, 37, 78], by including LU and WU as other indicators to assess impact of the diet. To the best of our knowledge this is the first study that explores in detail differences in both consumption of SOP and EI across SES, integrating the indicators of LU and WU in this context. Additionally, this study used the most recent data from the DNFCS data set, ensuring the generalizability to the Dutch population. Food consumption data was collected through two non-consecutive 24-h dietary recalls, a method that provides detailed dietary data with reduced bias [79]. By integrating these data with LCA results, this study combines two reliable and precise sources to provide SES-specific insights, assisting policy-makers to target more healthy and sustainable diets in the Dutch population.

A limitation of the present study is that causal inference cannot be established due to its cross-sectional design [80]. Residual confounding factors including food environments, migration background, and food beliefs may also persist and influence the association between SES, SOP consumed and their associated EI [37, 81, 82]. Although the 24-h dietary recall is a commonly used and validated method to collect dietary consumption data, misreporting, underreporting, and overreporting, may still occur [83]. Since data of misreporting was unavailable, no adjustments were made and therefore may have influenced the estimation of environmental impact.

Other important limitation is that only self-reported education level is available to define SES. Although education level has been considered a good proxy for SES [84], a measure composed by education level and income would be a stronger representation of SES [85].

Another limitation is that data from the DNFCS 2019–2021 were collected during the COVID-19 pandemic, which may affect representativeness of dietary patterns. Lockdowns led to shifts in dietary patterns, with individuals reporting more “unhealthy diets”, attributed to changes in daily routines, increased remote work and more reliance on food delivery services [42]. Although cereals contribute considerably to overall protein intake in the Dutch population [3, 33, 34], the present analysis focused on food groups commonly recognized as protein sources (e.g. meat, dairy, fish, legumes, eggs, nuts, and meat substitutes) due to their higher protein density and relevance to discussions on protein transition and environmental impact [8, 86]. Future studies could incorporate cereal products to provide a more comprehensive view of plant-based protein intake in the Netherlands.

The present study analyzed red meat as a single category (including beef, pork, veal, etc.) following the GloboDiet classification. As current evidence suggests that ruminant meats (e.g. beef and lamb) have substantially higher environmental impact than pork [32, 87], future research with larger samples or detailed dietary data could distinguish these subgroups to have a more granular understanding of the environmental impact of different red meats across SES groups to improve comparability with other studies and for potential meta-analyses.

Finally, the intake of SOP was expressed in grams of product consumed per day, which does not account for differences in protein content across foods. Future research could explore these differences, including distinguishing between types of dairy products (e.g., cheese, milk, yogurt) to assess the robustness of these findings.

## Conclusions

This study showed differences in SOP consumed across SES groups in The Netherlands and the associated EI indicated by GHG emissions, LU, and WU. Individuals with low SES consumed more ABPs, including red, processed meats, and dairy; and showed higher GHG emissions and LU, mainly driven by red and processed meat intake. In contrast, individuals with high SES consumed more PBPs, especially nuts and seeds, as well as fish. Total WU was higher in the high SES group, mainly attributed to their greater consumption of nuts and seeds. These findings highlight the relevance of considering socioeconomic disparities that could inform strategies to support a transition toward healthier and more sustainable diets.

## Supplementary Information

Below is the link to the electronic supplementary material.

Supplementary Material 1

## Data Availability

Data of the Dutch National Food Consumption Survey 2019–2021 can be requested for at https://www.rivm.nl/en/dutch-national-food-consumption-survey/data-on-request (Accessed on 15, September, 2023). Primary environmental data of food products can be found at: https://www.rivm.nl/voedsel-en-voeding/duurzaam-voedsel/database-milieubelasting-voedingsmiddelen.
