# Supplementary material for: Differences in plant- and animal-based protein sources consumed across socioeconomic groups in The Netherlands and their associated environmental impact
Source: Eur J Nutr. 2026 Mar 17;65(3):94. doi: 10.1007/s00394-026-03940-w (PMC12995988; doi:10.1007/s00394-026-03940-w)
Supplement: Supplementary file 1 — Supplementary Material 1 [file 394_2026_3940_MOESM1_ESM.docx]

Differences in plant- and animal-based protein sources consumed across socioeconomic groups in The Netherlands and their associated environmental impact

Hector A. Lopez Mariaca ^1^*, Christa Blokhuis ^1,2^ and Yinjie Zhu ^1,3^

1. Consumption and Healthy Lifestyles chair group, Wageningen University & Research, Hollandseweg 1, 6706 KN, Wageningen, The Netherlands; hectorandres28@gmail.com (H.A.L.M.); christa.blokhuis@wur.nl (C.B.); yinjie.zhu@wur.nl (Y.Z.)
2. Information Technology group, Wageningen University & Research, Hollandseweg 1, 6706 KN, Wageningen, the Netherlands.
3. National Institute for Public Health and the Environment, Antonie van Leeuwenhoeklaan 9, 3721 MA, Bilthoven, The Netherlands.

* Correspondence: yinjie.zhu@wur.nl: Tel: +31 317 488 270

**Supplementary file**

**Supplementary Table S1. Environmental impact of different SOP**

| Group product (kg) | GHG emissions  kg CO2 eq | LU  m^2^ | WU  L |
| --- | --- | --- | --- |
| Legumes | 2.53 | 3.05 | 60 |
| Nuts and seeds | 4.16 | 8.04 | 266 |
| Dairy | 5.06 | 2.11 | 40 |
| Red meat | 22.20 | 12.31 | 19 |
| Poultry | 12.21 | 8.38 | 17 |
| Processed meat | 13.48 | 8.29 | 12 |
| Meat substitutes | 4.14 | 2.83 | 7 |
| Fish | 8.44 | 1.87 | 5 |
| Eggs | 4.32 | 3.79 | 11 |

Average EI derived from different SOP according to the “Database for the environmental impact of food products” (*Database Milieubelasting voedingsmiddelen*).

**Supplementary Table S2.** **Total EI from consumption of SOP across SES groups.**

| **Environmental impact** | | **Total** | **Low SES** | **Middle SES** | **High SES** | ***p*** |
| --- | --- | --- | --- | --- | --- | --- |
|  |  | **(n = 1746)** | **(n = 445)** | **(n = 632)** | **(n = 669)** |  |
| Total GHG | 3.13 (2.16 – 4.20) | | 3.36 (2.39 - 4.51) | 3.23 (2.19 - 4.23) | 2.88 (1.94 - 3.99) | < 0.001 |
| GHG ABPs | 3.03 (2.05 – 4.12) | | 3.32 (2.35 - 4.48) | 3.15 (2.10 - 4.14) | 2.81 (1.79 - 3.91) | < 0.001 |
| GHG PBPs | 0.04 (0 – 0.15) | | 0.01 (0 - 0.10) | 0.04 (0 - 0.14) | 0.06 (0 - 0.18) | < 0.001 |
| GHG PBPs Ratio | 0.01 (0 – 0.05) | | 0 (0 - 0.03) | 0.01 (0 - 0.04) | 0.02 (0 - 0.07) | < 0.001 |
| Total LU | 1.66 (1.18 – 2.21) | | 1.75 (1.33 - 2.32) | 1.72 (1.19 - 2.27) | 1.53 (1.07 - 2.10) | < 0.001 |
| LU ABPs | 1.52 (1.03 – 2.08) | | 1.64 (1.20 - 2.20) | 1.59 (1.05 - 2.10) | 1.36 (0.87 - 1.90) | < 0.001 |
| LU PBPs | 0.08 (0 – 0.22) | | 0.01 (0 - 0.18) | 0.07 (0 - 0.21) | 0.12 (0 - 0.27) | < 0.001 |
| LU PBPs Ratio | 0.05 (0 – 0.14) | | 0.01 (0 - 0.09) | 0.04 (0 - 0.14) | 0.08 (0 - 0.19) | < 0.001 |
| Total WU | 46.94 (27.72 – 86.76) | | 44.45 (28.63 - 71.68) | 44.12 (28.07 - 87.78) | 50.69 (27.33 - 94.18) | 0.126 |
| WU ABPs | 27.44 (18.45 – 36.71) | | 29.53 (21.43 - 39.17) | 28.69 (18.61 - 36.99) | 25.29 (16.23 - 34.47) | < 0.001 |
| WU PBPs | 6.65 (0 – 59.02) | | 1.22 (0 - 39.90) | 5.52 (0 - 59.14) | 19.95 (0 - 67.78) | < 0.001 |
| WU PBPs Ratio | 0.24 (0 – 0.70) | | 0.05 (0 - 0.62) | 0.19 (0 - 0.69) | 0.46 (0 - 0.75) | < 0.001 |

Values are median (interquartile range). GHG (Greenhouse gas) kg CO₂-eq/d, GHG ABPs (Greenhouse gas emissions derived from Animal-based protein sources), GHG PBPs (Greenhouse gas emissions derived from Plant-based protein sources) GHG PBPs Ratio (GHG PBPs/Total GHG), LU (Land Use) m² crop eq/d, LU ABPs (Land Use derived from Animal-based protein sources), LU PBPs (Land Use derived from Plant-based protein sources) LU PBPs Ratio (LU PBPs/Total LU), WU (Water Use) L/d, WU ABPs (Water Use derived from Animal-based protein sources), WU PBPs (Water Use derived from Plant-based protein sources), WU PBPs Ratio (WU PBPs/Total WU), SES (Socioeconomic status).

**Supplementary Table S3. Modification effect of sex in the association of SES with different SOP consumed in the study population.**

| SES | Total protein (g/d) | | SES*Sex | | Animal-based protein (g/d) | | SES*Sex | |
| --- | --- | --- | --- | --- | --- | --- | --- | --- |
|  | β (ΣE) | *p* | β (ΣE) | *p* | β (ΣE) | *p* | β (ΣE) | *p* |
| Male |  |  | -2.36 (0.99) | 0.018 |  |  | -2.39 (1.08) | 0.027 |
| High | 1 | |  |  | 1 | |  |  |
| Middle | 0.97 (1.41) | 0.492 |  |  | 1.35 (1.51) | 0.371 |  |  |
| Low | 0.50 (1.66) | 0.762 |  |  | 3.15 (1.77) | **0.074** |  |  |
| Female |  |  |  |  |  |  |  |  |
| High | 1 | |  |  | 1 | |  |  |
| Middle | 2.65 (1.18) | **0.025** |  |  | 4.94 (1.29) | **< 0.001** |  |  |
| Low | 3.79 (1.31) | **0.003** |  |  | 6.68 (1.44) | **< 0.001** |  |  |

Multivariate linear regression analysis. Models adjusted for age, calorie intake, BMI, smoking, alcohol consumption and PA. Bolded values represent significant results (*p-*value < 0.05). ΣΕ (Standard error), SES (Socioeconomic status).

**Supplementary Table S4. Modification effect of sex in the association of SES with consumers of legumes in the study population.**

| SES | Legumes (yes) n (%) | | SES*Sex | |
| --- | --- | --- | --- | --- |
|  | OR (95% CI) | *p* | β (ΣE) | *p* |
| Male |  |  | -2.36 (0.99) | 0.018 |
| High | 1 | |  |  |
| Middle | 0.94 (0.58 - 1.51) | 0.787 |  |  |
| Low | 1.69 (1.02 - 2.80) | 0.042 |  |  |
| Female |  |  |  |  |
| High | 1 | |  |  |
| Middle | 0.63 (0.38 - 1.04) | 0.073 |  |  |
| Low | 0.47 (0.25 - 0.85) | **0.014** |  |  |

Multivariate logistic regression analysis. Models adjusted for age, calorie intake, BMI, smoking, alcohol consumption and PA. Bolded values represent significant results (*p-*value < 0.05). OR (Odds Ratios), CI (Confidence intervals), SES (Socioeconomic status.

**Supplementary Table S5. Modification effect of sex in the association of socio-economic status (SES) with GHG emissions derived from different SOP consumed in the study population.**

|  | Total GHG emissions | | SES*Sex | | ABP GHG | | SES*Sex | | PBP GHG Ratio | | SES*Sex | |
| --- | --- | --- | --- | --- | --- | --- | --- | --- | --- | --- | --- | --- |
|  | β (ΣΕ) | *p* | β (ΣΕ) | *p* | β (ΣΕ) | *p* | β (ΣΕ) | *p* | β (ΣΕ) | *p* | β (ΣΕ) | *p* |
| Male |  |  | -0.19 (0.09) | **0.031** |  |  | -0.20 (0.09) | **0.024** |  |  | -0.02 (0.00) | **0.004** |
| High | 1 | |  |  | 1 | |  |  | 1 | |  |  |
| Middle | 0.18 (0.12) | 0.147 |  |  | 0.19 (0.13) | 0.129 |  |  | -0.00 (0.00) | 0.716 |  |  |
| Low | 0.43 (0.14) | **0.003** |  |  | 0.46 (0.15) | **0.002** |  |  | -0.00 (0.00) | 0.328 |  |  |
| Female |  |  |  |  |  |  |  |  |  |  |  |  |
| High | 1 | |  |  | 1 | |  |  | 1 | |  |  |
| Middle | 0.46 (0.10) | **<0.001** |  |  | 0.50 (0.10) | **<0.001** |  |  | -0.04 (0.01) | **<0.001** |  |  |
| Low | 0.66 (0.11) | **<0.001** |  |  | 0.71 (0.11) | **<0.001** |  |  | -0.04 (0.01) | **<0.001** |  |  |

Multivariate linear regression analysis. Models adjusted for age, calorie intake, BMI, smoking, alcohol consumption and PA. Bolded values represent significant results (*p-*value < 0.05). ΣΕ (Standard error), SES (socioeconomic status), ABP (animal-based protein sources), PBP (plant-based protein sources), GHG (greenhouse gas),

**Supplementary Table S6. Modification effect of sex in the association of socio-economic status (SES) with LU derived from different SOP consumed in the study population.**

|  | Total LU | | SES*Sex | | ABP LU | | SES*Sex | | PBP LU Ratio | | SES*Sex | |
| --- | --- | --- | --- | --- | --- | --- | --- | --- | --- | --- | --- | --- |
|  | β (ΣΕ) | *p* | β (ΣΕ) | *p* | β (ΣΕ) | *p* | β (ΣΕ) | *p* | β (ΣΕ) | *p* | β (ΣΕ) | *p* |
| Male |  |  | -0.10 (0.04) | **0.022** |  |  | -0.10 (0.04) | **0.017** |  |  | 0.02 (0.00) | **0.003** |
| High | 1 | |  |  | 1 | |  |  | 1 | |  |  |
| Middle | 0.09 (0.06) | 0.128 |  |  | 0.11 (0.06) | 0.081 |  |  | -0.01 (0.01) | 0.347 |  |  |
| Low | 0.22 (0.07) | **0.002** |  |  | 0.27 (0.07) | **<0.001** |  |  | -0.02 (0.01) | 0.075 |  |  |
| Female |  |  |  |  |  |  |  |  |  |  |  |  |
| High | 1 | |  |  | 1 | |  |  | 1 | |  |  |
| Middle | 0.24 (0.05) | **<0.001** |  |  | 0.29 (0.05) | **<0.001** |  |  | -0.06 (0.01) | **<0.001** |  |  |
| Low | 0.33 (0.05) | **<0.001** |  |  | 0.40 (0.05) | **<0.001** |  |  | -0.07 (0.02) | **<0.001** |  |  |

Multivariate linear regression analysis. Models adjusted for age, calorie intake, BMI, smoking, alcohol consumption and PA. Bolded values represent significant results (*p-*value < 0.05). ΣΕ (Standard error), SES (Socioeconomic status), ABP (animal-based protein sources), PBP (plant-based protein sources), LU (land ue).

**Supplementary Table S7. Modification effect of sex in the association of socio-economic status (SES) with WU derived from different SOP consumed in the study population.**

|  | ABP WU | | SES*Sex | |
| --- | --- | --- | --- | --- |
|  | β (ΣΕ) | *p* | β (ΣΕ) | *p* |
| Male |  |  | -1.83 (0.76) | **0.015** |
| High | 1 | |  |  |
| Middle | 1.64 (1.08) | 0.127 |  |  |
| Low | 4.01 (1.26) | **0.001** |  |  |
| Female |  |  |  |  |
| High | 1 | |  |  |
| Middle | 4.69 (0.89) | **<0.001** |  |  |
| Low | 6.26 (0.99) | **<0.001** |  |  |

Multivariate linear regression analysis. Models adjusted for age, calorie intake, BMI, smoking, alcohol consumption and PA. Bolded values represent significant results (*p-*value < 0.05). ΣΕ (Standard error), SES (Socioeconomic status), ABP (animal-based protein sources), WU (water use).
